# An Infant With Kawasaki Disease: A Case Report and Literature Review

**DOI:** 10.7759/cureus.65221

**Published:** 2024-07-23

**Authors:** Yifan Ren

**Affiliations:** 1 Department of Paediatrics, Shaoxing Keqiao Women and Children’s Hospital, Shaoxing, CHN

**Keywords:** coronary artery, case report, infant, complete kd, kawasaki disease

## Abstract

Kawasaki disease (KD) is the leading cause of acquired heart disease in children in developed countries. Delayed treatment can lead to coronary artery (CA) abnormalities, potentially causing myocardial ischemia, infarction, and death. Younger age is a risk factor for developing bilateral large CA aneurysms in KD patients. A one-and-a-half-month-old infant presented with fever and elevated inflammatory markers. Post-admission ceftriaxone injections were ineffective. Subsequently, the patient experienced recurrent high fevers, accompanied by rashes, erythema, and induration of the palms and soles, erythema, swelling at the Bacillus Calmette-Guerin (BCG) scar site, cracked lips, and conjunctival hyperemia, all of which were indicative of KD. Intravenous immunoglobulin (IVIG) and aspirin were administered on the third day of fever. Follow-ups at one, three, six, and 12 months post discharge revealed normal findings. This case demonstrates that even very young infants can develop complete KD, and early treatment can prevent CA complications.

## Introduction

Due to passive immunity (before six months of age) and protective antibody responses to common antigens (after five years of age), Kawasaki disease (KD) manifests as a self-limited, systemic inflammatory vasculitis primarily affecting children aged six months to five years [1]. Studies indicate that infants under six months have a greater incidence of incomplete KD and are at higher risk of resistance to intravenous immunoglobulin (IVIG) treatment and cardiac complications [2,3]. A case of complete KD in an infant is documented herein, with a favorable prognosis. This case underscores that even very young infants can develop complete KD and emphasizes the importance of early treatment in preventing coronary artery (CA) damage.

## Case presentation

A one-and-a-half-month-old boy, previously in good health, was admitted to our hospital with a fever lasting 1.5 days (39.0 °C), along with nasal congestion, a runny nose, and an occasional cough. No additional symptoms of KD were observed upon admission (day 1.5). The child had no prior exposure to coronavirus 19 (COVID-19) and tested negative for COVID-19 nucleic acid. The initial blood test results revealed that the C-reactive protein (CRP) was marginally higher than usual and that the white blood cell count (WBC) was high, mostly neutral (Table 1). Vital indicators were within the normal range during the initial physical examination. The pharynx exhibited swelling and slight redness at the site of the left Bacillus Calmette-Guerin (BCG) injection, with other physical examination findings being normal. Upon admission, ceftriaxone (80 mg/kg, once a day (QD)) was administered to address the bacterial infection.

**Table 1:** Laboratory investigations of the patient WBC: white blood cell count; N%: neutrophil percentage; L%: lymphocyte percentage; Hb: hemoglobin; PLT: platelets; CRP: C-reactive protein; BUN: urea nitrogen; SCR: creatinine; UA: uric acid; ALT: alanine aminotransferase; AST: aspartate transaminase; ESR: erythrocyte sedimentation rate

Test	Results of the first blood test	Results of the second blood test	Results of the third blood test	Results of the fourth blood test	Reference range
WBC	21.3	14.6	28.8	8.8	4.0-10.0x 10^9^/L
N%	51.6	55.6	64.9	28.4	40-75%
L%	26.3	34.4	24.1	56.3	40-60%
Hb	117	124	128	105	110-150g/L
PLT	419	379	486	603	100-300x 10^9^/L
CRP	25.2	42.7	76.7	15.6	0-8mg/L
BUN		2.75			1.43-7.14mmol/L
SCR		23			44-97mmol/L
ALT		17			9-50U/L
AST		26			15-40U/L
Total protein		58			65-85g/L
Albumin		37.6			38-55g/L
UA		161			208-428mmol/L
ESR			51.3		0-15mm/H

**Figure 1:**
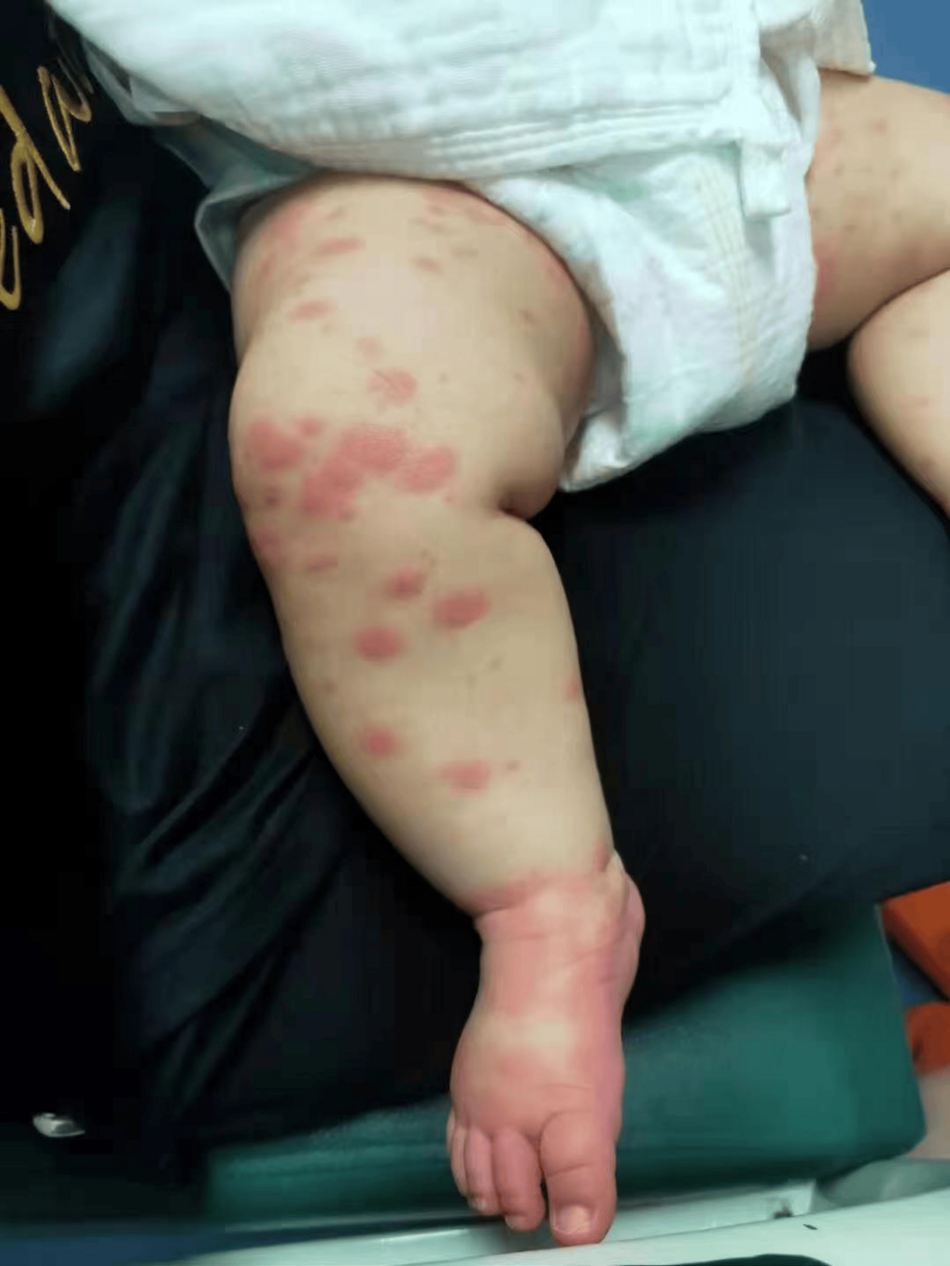
The patient’s limbs were largely affected by sporadic red rashes, and the soles of his feet were swollen and red.

**Figure 2:**
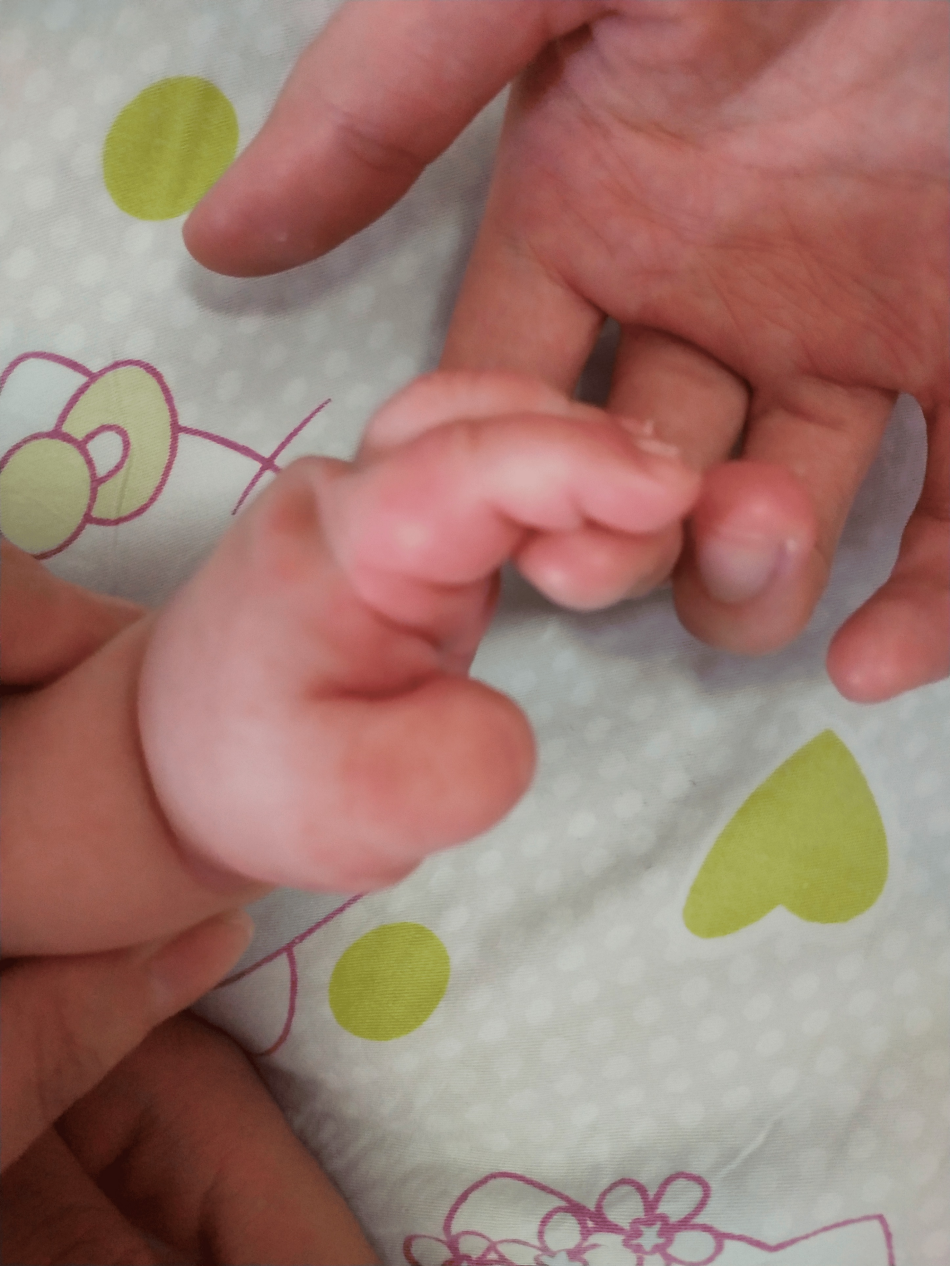
The patient had red, swollen palms.

The patient’s first day of hospitalization was marked by four episodes of high fever, sporadic red rashes on the face, chest, back, and limbs (Figure 1), swollen red soles of the feet and palms (Figure 2), and red, inflamed skin near the BCG vaccine site. Normal urination suggested no urinary tract infection. An ultrasound examination revealed no visibly enlarged cervical lymph nodes. Echocardiography indicated normal inner diameters of the left and right CAs (1.7 mm and 1.8 mm, respectively) without dilatation. White blood cell counts were lower, while CRP was higher in the second blood test. Figure 1 presents more findings. Ceftriaxone treatment for bacterial infections continued, while CRP levels continued to rise. On the second day of admission (day three of fever), the patient developed a fever every three to four hours, peaking at 40.1 °C. The anterior fontanelle was soft, with a passive reaction. Dry lips and conjunctival hyperemia were observed, but no purulent discharge was present. Antigen testing for adenovirus, parainfluenza virus 1.2.3, respiratory syncytial virus, influenza A, and influenza B yielded negative results.

**Figure 3:**
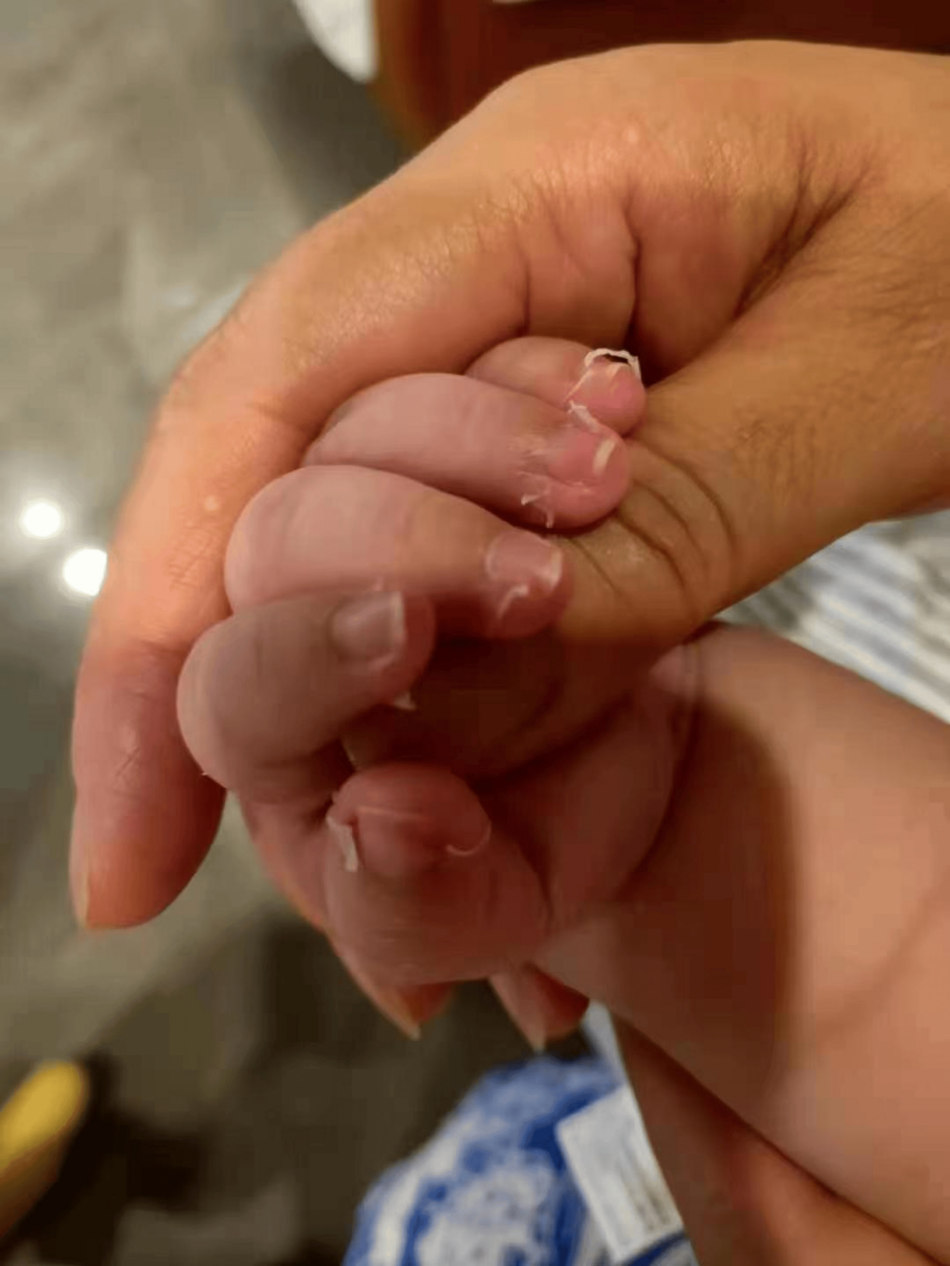
Two days after being discharged, the patient’s fingertips molted.

The third blood examination's results revealed that the erythrocyte sedimentation rate (ESR) was much higher than normal, and the WBC and CRP were highly elevated (Table 1). The patient was diagnosed with KD. Treatment included oral administration of aspirin (37.5 mg/kg/d), IVIG at a dose of 2 g/kg, and cessation of ceftriaxone. Subsequently, the fever subsided, the rash disappeared, and the redness and puffiness of the BCG scar reduced; however, the patient’s lips remained chapped on the third day of admission. By the fifth day after admission, the temperature normalized, the rash vanished, the BCG scar ceased to be red, the lips were no longer chapped, and ocular conjunctiva congestion resolved. A repeat echocardiogram revealed unchanged inner diameters of the left and right CAs (1.8 mm each) without dilatation. White blood cells and CRP were considerably lower, according to the findings of the fourth blood test (Table 1). Aspirin dosage was reduced to 3 mg/kg/d. After two days, fingertip peeling occurred (Figure 3), and the patient was discharged on the seventh day of admission. Aspirin was discontinued after six weeks. Subsequent follow-ups at one month, three months, six months, and one year postdischarge revealed normal physical examination findings, electrocardiogram (ECG), and echocardiography results.

## Discussion

We reviewed relevant literature and Table *2* presents a summary.

**Table 2:** Treatment and outcomes of infant Kawasaki disease patients

Case source	Age	Sex	Evolution	Complications	Kawasaki disease (KD)	Time to apply intravenous immunoglobulin (IVIG) after initial illness	Echocardiogram	Treatment
Rim Kasem Ali Sliman et al. [4]	8 years	Female	4 weeks	Retropharyngeal abscess	Complete Kawasaki disease KD	9 days	Normal	IVIG
Chu et al. [5]	10 months	Male	7 months	Peripheral systemic artery aneurysms and thrombotic events	Complete KD	10 days	Giant bilateral multiple coronary artery aneurysms (CAA)	IVIG, aspirin, dipyridamole
Kawamura et al. [2]	1 month	Male	7 months	Severe pneumonia	Incomplete KD	3 days	Bilateral CAA	IVIG, aspirin, cyclosporine
Rajandran et al. [3]	3.5 months	Female	4.5 months	Marked and unexplained irritability	Incomplete KD	4 weeks	Bilateral CAA	IVIG, aspirin
Current case	1 month and 15 days	Male	1 year	Nasal congestion, a runny nose, and occasional cough	Complete KD	3 days	Normal	IVIG, aspirin

One month was the youngest age at which KD first appeared (Table 2) [2]. In contrast to our case, the infant in the study by Kawamura et al. [2] was diagnosed with incomplete KD with CA dilatation after presenting with acute pneumonia as the initial symptom. In addition to IVIG and aspirin, cyclosporin was also employed in the treatment. The exact cause of KD remains unknown. However, it is believed to be an immune-mediated disease triggered by infection in individuals with a genetic predisposition [6-8]. Early in the illness, the baby in our case experienced respiratory symptoms. Though future research will focus on this area, the cause of this case cannot currently be ascertained because our facility is currently unable to complete the genetic testing linked to KD.

Distinguishing recurrent high fever and increased signs of infection in children requires consideration of various conditions, such as urinary tract infection, suppurative meningitis, sepsis, and multisystem inflammatory syndrome in children (MIS-C). Urinalysis results ruled out urinary tract infection, while the absence of vomiting, consciousness disorder, and convulsions ruled out purulent meningitis. Inexplicable signs of sepsis were observed in the patient, including redness and swelling at the BCG vaccine site, swollen hands and feet, and chapped lips, without a clear bacterial infection focus. Antibacterial treatment proved ineffective, and the sepsis diagnosis lacked support. In April 2020, MIS-C emerged in regions with high COVID-19 rates. Epidemiological evidence suggests COVID-19 as the cause of MIS-C, characterized by shock, rash, cheilitis, and conjunctivitis, similar to KD [9]. The child had never been exposed to COVID-19 and was negative for COVID-19 nucleic acid. This evidence does not support the diagnostic criteria for “classic” KD, which involves fever for at least five days along with at least four of the five principal clinical features. However, experienced clinicians may diagnose KD with three days of fever [10]. The diagnosis in this case met the “classic” criteria.

Seasonal variations in KD incidence are well documented [11, 12], with unclear causality related to climatic changes or other seasonal effects. Specific wind conditions and temperature and precipitation variations have been implicated [13], with KD incidence peaking in January and June in Japan [14], consistent with this case occurring in July. Recent evidence highlights a significant rate of CA aneurysms in infants under six months old [12]. Timely IVIG administration in this case likely prevented CA damage.

## Conclusions

Kawasaki disease is a self-limited inflammatory vasculitis that typically affects children between the ages of six months and five years. Infants under the age of six months are more likely to experience incomplete KD, as well as cardiac problems and resistance to IVIG treatment. This instance highlights the possibility of complete KD developing in even very young neonates. When unexplained sepsis symptoms, such as recurrent fever, poor response to antibiotics, rash, swollen extremities, and localized inflammation at the BCG vaccination site, are evident during local epidemic seasons, investigation of KD is needed, even in tiny babies. Early IVIG treatment helps lessen CA harm.
